# Biochemical and Metabolic Changes in Arsenic Contaminated* Boehmeria nivea* L.

**DOI:** 10.1155/2016/1423828

**Published:** 2016-02-28

**Authors:** Hussani Mubarak, Nosheen Mirza, Li-Yuan Chai, Zhi-Hui Yang, Wang Yong, Chong-Jian Tang, Qaisar Mahmood, Arshid Pervez, Umar Farooq, Shah Fahad, Wajid Nasim, Kadambot H. M. Siddique

**Affiliations:** ^1^Department of Environmental Engineering, School of Metallurgy and Environment, Central South University, Changsha, Hunan 410083, China; ^2^National Engineering Research Center for Control and Treatment of Heavy Metal Pollution, Changsha 410083, China; ^3^Department of Environmental Sciences, COMSATS Institute of Information Technology, Abbottabad 22060, Pakistan; ^4^Department of Chemistry, COMSATS Institute of Information Technology, Abbottabad 22060, Pakistan; ^5^College of Plant Science and Technology, Huazhong Agricultural University, Wuhan, Hubei 430070, China; ^6^Department of Environmental Sciences, COMSATS Institute of Information Technology (CIIT), Vehari, Pakistan; ^7^The UWA Institute of Agriculture, The University of Western Australia, Perth, WA 6009, Australia

## Abstract

Arsenic (As) is identified by the EPA as the third highest toxic inorganic contaminant. Almost every 9th or 10th human in more than 70 countries including mainland China is affected by As. Arsenic along with other toxins not only affects human life but also creates alarming situations such as the deterioration of farm lands and desertion of industrial/mining lands. Researchers and administrators have agreed to opt for phytoremediation of As over costly cleanups.* Boehmeria nivea* L. can soak up various heavy metals, such as Sb, Cd, Pb, and Zn. But the effect of As pollution on the biology and metabolism of* B. nivea* has been somewhat overlooked. This study attempts to evaluate the extent of As resistance, chlorophyll content, and metabolic changes in As-polluted (5, 10, 15, and 20 mg L^−1^ As)* B. nivea* in hydroponics. Toxic effects of As in the form of inhibited growth were apparent at the highest level of added As. The significant changes in the chlorophyll, electrolyte leakage, and H_2_O_2_, significant increases in As in plant parts, catalase (CAT), and malondialdehyde (MDA), with applied As revealed the potential of* B. nivea* for As decontamination. By employing the metabolic machinery of* B. nivea*, As was sustainably removed from the contaminated areas.

## 1. Introduction

Ramie (*Boehmeria nivea*), commonly known as China grass, is an important fiber crop which has been widely cultivated and distributed in China. It is a perennial plant with at least three harvests per year [1]. The principal end product of* B. nivea* is textile grade fiber, famous for its fine characteristics in textile industries [2]. The leaf and the root extracts of the plant have antimicrobial, anti-inflammatory, antioxidant, and hepatoprotective properties [3]. Ramie grows in the wild and is known to colonize both active and abandoned metal mine sites. It is capable of accumulating certain amounts of toxins such as Sb, Cd, and Hg [4, 5]. Other studies have shown that ramie can also tolerate certain amounts of heavy metals such as mercury, lead [6], cadmium [7, 8], and arsenic [1].

Across the globe, centuries of unsustainable activities have resulted in severe heavy metal contamination which has damaged aquatic and terrestrial environments [9]. Arsenic (As) is one of these reported toxins and is carcinogenic. It is a ubiquitous element and its toxicity in the environment is a global issue. The increasing As contamination in water, soils, and crops in numerous countries such as India, Bangladesh, Cambodia, Laos, Myanmar, China, Taiwan, United States, Vietnam, Thailand, and Europe is well reported [9, 10]. The health problems associated with chronic exposure to As are diabetes, cancer, poisoning, pathogenic potential of bacteria or fecal coliform, and blood stream infections. In China, high arsenic groundwater has been observed in the Datong basin of Shanxi Province, Hetao basin of Inner Mongolia, Xinjiang and Taiwan Provinces. In these regions, approximately 18.5 million people are at risk of exposure to high arsenic groundwater [11].

Colonization of* B. nivea* and accumulation of heavy metals such as Sb, Cd, Pb, and Zn in metal-contaminated regions have been reported.* B. nivea* has a high tolerance to As contamination [12]. The effect of As pollution on the biology of* B. nivea*, the potential tolerance of* B. nivea* to As pollution, and the metabolic changes in* B. nivea* under the reported ranges of As contamination has not been documented. Based on the above review [12], the objectives of the current study were to evaluate plant growth, metabolic changes, and the ability of* B. nivea* L. to uptake and accumulate As in an attempt to determine the effects of As on chlorophyll content, antioxidative systems, and lipid membrane peroxidation in order to better understand the cellular basis of As tolerance in* B. nivea*.

## 2. Materials and Methods

### 2.1. Plant Materials and Growth Conditions

Young shoot cuttings (14 cm) of* B. nivea* plants were collected from an active and abandoned mining area in Xikuangshan, Hunan Province, China (29°N, 120°E). The cuttings were planted in sand for root initiation and then transferred to 1/2 strength Hoagland solution until they reached a height of 30 cm (40 days).

### 2.2. Hydroponic Experiment

After 40 days, the plants were transferred to 1/2 strength Hoagland solution (2 L) spiked with 0, 5, 10, 15, or 20 mg L^−1^ of arsenic. Arsenic (As) was applied as NaAsO_2_ (As-III, 100% purity). Each treatment was replicated three times. After 14 days of As exposure, the roots were immersed in 20 mM Na_2_-EDTA for 30 min to remove the As adsorbed to the roots, and the entire plant was rinsed three times with deionized water. The roots, stems, and leaves were separated. For further analysis, some of the fresh top leaves were frozen at −80°C and some were dried at 70°C. The remaining Hoagland solution was filtered and refrigerated for later As analysis. The stress tolerance index of As-contaminated* B. nivea* was calculated according to Yang et al. and Ismail et al. [1, 13].

The stress tolerance index (%) was calculated using the following formula:

(1) Stress  tolerance  index=Shoot  length  of  stress  plantShoot  length  of  control  plant×100.

### 2.3. Metabolism of* B. nivea*

#### 2.3.1. Chlorophyll Analysis

The 6th fully expanded leaf from the top of each plant was used to measure chlorophyll a, chlorophyll b, and total chlorophyll (chl (a + b)). Chlorophyll a, chlorophyll b, and chl (a + b) were analyzed according to the methods of Arnon and Huang et al. [14, 15] and estimated using the formulas of Ehsan et al. and Metzner et al. [16, 17]:

(2) Chlorophyll  a  μg mL−1=10.3×E663−0.98×E644Chlorophyll  b  μg mL−1=19.7×E644−3.87×E663Total  chlorophyll=Chlorophyll  a+Chlorophyll  b.

#### 2.3.2. Assessment of Antioxidants

Physiological measurements were performed on the 5th leaf from the top of plants growing in well-watered As conditions. Approximately 0.2 g of fresh tissue was homogenized in a precooled mortar with 5 mL of 50 mmol L^−1^ precooled phosphate buffer (pH 7.8). The homogenate was centrifuged at 11,000 g for 20 min at 4°C. The supernatant (i.e., the enzyme extract) was used to determine enzyme activities—superoxide dismutase (SOD), peroxidase (POD), catalase (CAT), and malondialdehyde content (MDA) [18, 19].

#### 2.3.3. Assessment of Electrolyte Leakage and Hydrogen Peroxide Levels

Electrolyte leakages in 5 mm long fragments of fully expanded leaves were determined. The fragment tubes were incubated in a water bath at 32°C for 2 hours with the initial electrical conductivity (EC) of the medium, EC_1_, noted. The samples were autoclaved at 121°C for 20 min to discharge the electrolytes and then cooled to 25°C. The final EC_2_ was measured [20, 21]. Electrolyte leakage (EL) was calculated using the following formula:

(3) $$\begin{array}{c} EL=\left(\;\frac{EC_{1}}{EC_{2}}\;\right)\times 100. \end{array}$$

H_2_O_2_ contents were assayed colorimetrically as documented by Jana and Choudhuri and Shakoor et al. [22, 23]. The hydrogen peroxide was extracted by homogenizing 0.05 g leaf tissues with 3 mL of phosphate buffer (50 mM, pH 6.5). The homogenate was centrifuged at 6,000 g for 25 min. To measure H_2_O_2_ content, 2.5 mL of the extracted solution was mixed with 1 mL of 0.1% titanium sulfate (Ti(SO_4_)_2_) in 20% (v/v) H_2_SO_4_. The mixture was centrifuged at 6,000 g for 15 min. The intensity of the yellow color supernatant was analyzed at 410 nm. The H_2_O_2_ content was calculated by applying an extinction coefficient of 0.28 mmol^−1 ^cm^−1^.

#### 2.3.4. Assessment of Relative Water Contents (RWC)

The RWC was determined using the methods of Huang et al. and Yamasaki and Dillenburg [15, 24]. The fresh mass (FM) of leaves was immersed in distilled water for 12 h to determine turgid mass (TM). Leaves were then dried at 70°C for 48 h to determinate dry mass (DM). The RWC was calculated as follows:

(4) $$\begin{array}{c} RWC=\left[\;\frac{\left(FM-DM\right)}{\left(TM-DM\right)}\;\right]\times 100. \end{array}$$

### 2.4. Arsenic (As) Analysis

The dried plant samples were ground, sieved (1 mm), and digested with HNO_3_ : HClO_4_ (4 : 1, v/v). The As concentration in plant parts was analyzed using Induced Couple Plasma-Optical Emission Spectrometer (ICP-OES) (Perkin Elmer, Precisely, Shelton, CT 06484, USA, Optima*™* 5300 DV Spectrometer). For accuracy of the digestion and analytical method, a blank sample (4 mL HNO_3_ + 1 mL HClO_4_) was also run with the samples.

### 2.5. Data Analysis

Analysis of variance (ANOVA) at a significance level of *P* < 0.05 was performed using the General Linear Model (GLM) in the SAS package. The LSD test and *t*-test were employed to compare significant differences between means for the treatments at *P* < 0.05. The results are expressed as means ± SD. Graphical analyses were carried out using Origin Pro 8.5.

## 3. Results and Discussion

### 3.1. Growth of* B. nivea*

Field surveys have reported the presence of healthy growing* B. nivea* plants in toxic metal-contaminated areas [8, 25], but only a few studies have reported As resistance in* B. nivea*. The higher tolerance of* B. nivea*, compared to other plant species, for toxic [26] and heavy metals [27], has been estimated and documented. The metabolic responses of* B. nivea* under specific As ranges, say between 10 and 250 mg kg^−1^ soil, have not been assessed.

**Figure 1:**
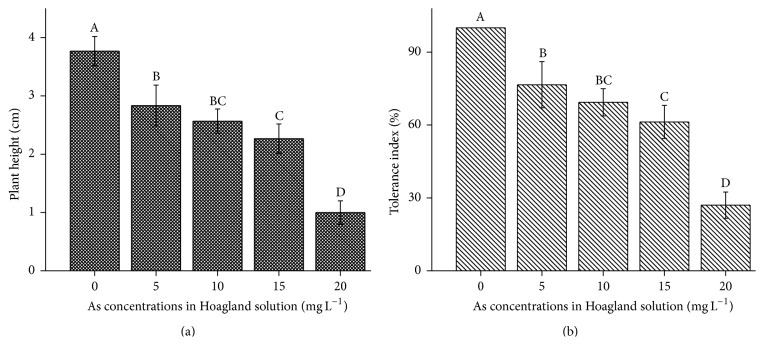
(a) Effect of As application, on the height of* Boehmeria nivea* L. (b) Effect of As application, on the tolerance index of* Boehmeria nivea*. Values followed by different uppercase letters are significantly different at P < 0.05, for treatments. Different letters indicate significant differences between treatments for each parameter, at significant level of 0.05. Values followed by the same letters for each parameter are not significantly different at the 0.05 level (least significant difference). Values in the graph are mean (*n* = 3); error bars are standard deviation (SD).

The As-contamination (hydroponic) treatments inhibited the growth of* B. nivea* more so as the contamination increased (Figure 1(a)). Plant height decreased with increasing As concentration. At 0 mg L^−1^ As, plant height ranged from 3.8 to 4 cm; the As treatments at 5, 10, 15, and 20 mg L^−1^ reduced plant height by 25, 32, 40, and 73% of the control, respectively. The As tolerance index of* B. nivea* significantly decreased (*P* < 0.05) as As concentration increased (Figure 1(b)) and ranged from 27 to 77%.

### 3.2. Metabolism of As-Contaminated* B. nivea* L

#### 3.2.1. Chlorophyll Content of* B. nivea* L

**Figure 2:**
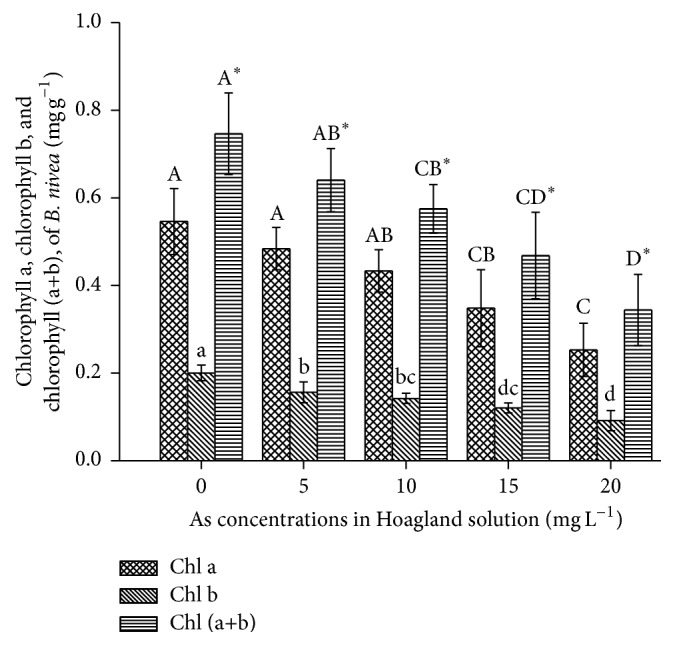
Effect of As application, on the chlorophyll of* Boehmeria nivea* L. Values followed by different uppercase letters are significantly different at P < 0.05, for treatments. Values followed by different lowercase letters are significantly different at *P* < 0.05, for treatments. Values followed by different uppercase letters^*∗*^ are significantly different at P < 0.05, for treatments. Different letters indicate significant differences between treatments for each parameter, at significant level of 0.05. Values followed by the same letters for each parameter are not significantly different at the 0.05 level (least significant difference). Values in the graph are mean (*n* = 3); error bars are standard deviation (SD).

The effect of applied As on chlorophyll content is presented in Figure 2. As the As concentration increased, all chlorophyll content measurements (chl a, chl b, and chl a + b) significantly decreased (*P* < 0.05) in* B. nivea* by 11–54%, 22–54% and 14–54%, respectively, relative to the control (Figure 2). Singh et al. [28] reported increased chlorophyll contents in* Pteris vittata* but decreased chlorophyll contents in* Pteris ensiformis* under As-induced stress.

#### 3.2.2. Activities of Antioxidant Enzymes in* B. nivea* L

**Figure 3:**
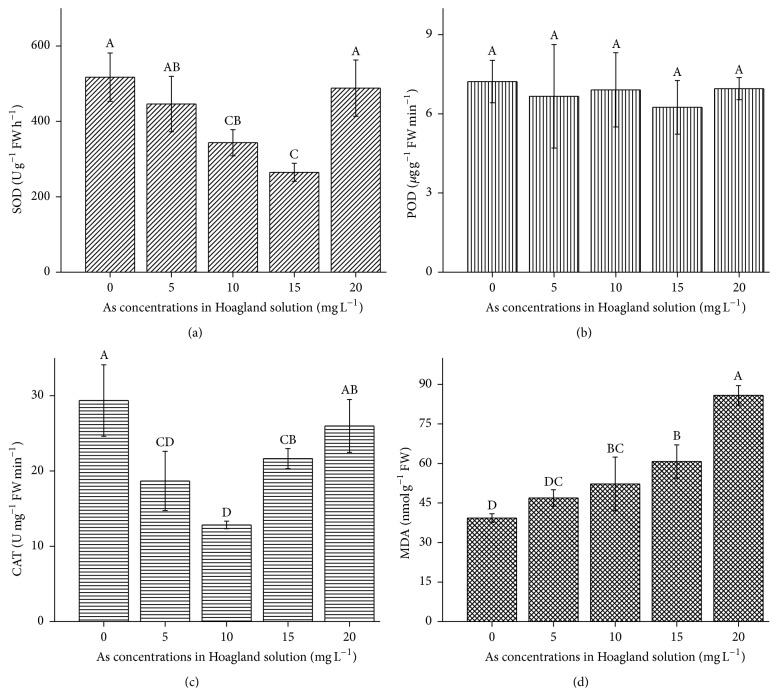
Effect of As application on antioxidant enzymes of* Boehmeria nivea*. (a): SOD; (b): POD; (c): CAT; (d): MDA. Values followed by different uppercase letters are significantly different at P < 0.05, for treatments. Different letters indicate significant differences between treatments for each parameter, at significant level of 0.05. Values followed by the same letters for each parameter are not significantly different at the 0.05 level (least significant difference). Values in the graph are mean ± SD (*n* = 3); error bars are SD.

Increasing As concentration had a significant (*P* < 0.05) effect on SOD, CAT, and MDA concentrations in* B. nivea* (Figures 3(a)–3(d)). Compared with the control, increasing the As concentration significantly decreased (*P* < 0.05) SOD concentration in* B. nivea*, except at the highest applied As (20 mg L^−1^), while there were a nonsignificant reduction in POD concentration and a gradual reduction in CAT concentration at 10 mg L^−1^, but a slight increase at 15 and 20 mg L^−1^. The MDA content in As-contaminated* B. nivea* showed a significant (*P* < 0.05) increasing trend with increasing As concentration.

In plants and living organisms, stress induces the generation of reactive oxygen species (ROS) which may cause oxidative damage to proteins and enzymes. Excessive ROS increases MDA, the last product of membrane liposome peroxidation, which suggests lipid membrane instability [29]. To reduce oxidative damage, plants initiate enzymatic and nonenzymatic antioxidant defense mechanisms, of which the synthesis of SOD, POD, and CAT is the most important. In* B. nivea*, the greatest increase in MDA, relative to the control, was at 20 mg L^−1^ As (Figures 3(a)–3(c)), demonstrating that POD and CAT are H_2_O_2_ scavengers in ramie. SOD, POD, and CAT activities had similar suppressive effects and enhanced trends with As addition, except for POD concentration at 10 mg L^−1^ As and CAT at 15 mg L^−1^ As. The critical-stage performance of antioxidants, in* B. nivea*, was at 20 mg L^−1^ As.

SOD contents in* B. nivea* decreased by approximately 1.2, 1.5, 2.0, and 1 times of the control at 5, 10, 15, and 20 mg L^−1^ As, respectively (Figure 3(a)). Based on this and the observed fluctuations, we conclude that SOD contributed to the tolerance of* B. nivea* to As contamination. At 5, 10, 15, and 20 mg L^−1^ As, POD content declined by approximately 1.1, 1.0, 1.2, and 1.0 times of the control, respectively (Figure 3(b)). Similar decreasing trends of SOD and POD contents in cadmium-stressed wheat (*Triticum durum*) and selenium-stressed ryegrass (*Lolium perenne*) have been reported [19]. A study by Saidi et al. [30] reported suppressed activities of SOD and POD in cadmium-contaminated bean plants, while Huang et al. [31] reported increased SOD and POD contents in hybrid ramie under increased salinity.

The CAT content in* B. nivea* decreased at 5, 10, 15, and 20 mg L^−1^ by 1.6-, 2.3-, 1.4-, and 1.1-fold less than control (Figure 3(c)). Silva et al. and Huang et al. [31, 32] reported decreasing CAT content in aluminum-exposed rye and salinity-stressed ramie. The decreased CAT activity with increasing As contamination confirms the role of CAT in quenching H_2_O_2_ and preventing oxidative damage in* B. nivea*.

Increasing the concentration of As in* B. nivea* increased MDA concentration from 1.12 times greater than control at 5 mg L^−1^ to 2.20 times greater than control at 20 mg L^−1^ (Figure 3(d)). The increase at 20 mg L^−1^ As suggests the role of MDA in lipid peroxidation and the maintenance of homeostasis of* B. nivea*. Increases in MDA activity below 20 mg L^−1^ As inhibited biomass production which is a clear indication of As tolerance of* B. nivea*. Our results of enhanced MDA and CAT activities with As addition agree with those of Feng et al. and Huang et al. [29, 31] who reported increased MDA and CAT activities in drought-stressed drought-resistant ramie cultivars, plants (ferns, rice, and maize) and hybrid ramie (*B. nivea*), respectively. Our results suggest that* B. nivea* is capable of alleviating oxidative stress and preventing lipid peroxidation under a specified range (5–15 mg L^−1^ As) of As contamination. The highest increase in MDA (2.20 times greater than control) at 20 mg L^−1^ confirms lipid peroxidation or damage to the plasma membrane which, in turn, inhibits plant growth.

#### 3.2.3. Electrolyte Leakage and Hydrogen Peroxide (H_2_O_2_) Levels in* B. nivea* L

**Figure 4:**
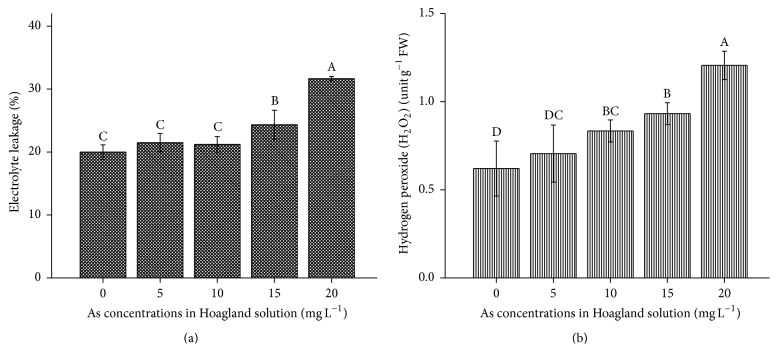
(a) Effect of As application, on the electrolyte leakage in* Boehmeria nivea* L. (b) Effect of As application, on the H_2_O_2_ in* Boehmeria nivea* L. Values followed by different uppercase letters are significantly different at P < 0.05, for treatments. Different letters indicate significant differences between treatments for each parameter, at significant level of 0.05. Values followed by the same letters for each parameter are not significantly different at the 0.05 level (least significant difference). Values in the graph are mean (*n* = 3); error bars are standard deviation (SD).

Solute leakage and H_2_O_2_ content increased in* B. nivea* with increasing As contamination (Figures 4(a) and 4(b)). The increases in electrolyte leakage and H_2_O_2_ from 5 to 20 mg L^−1^ As ranged from 1- to 1.5-fold and 1.13- to 2-fold greater than the control, respectively. The gradual increase in MDA, electrolyte leakage, and H_2_O_2_ from 5 to 15 mg L^−1^ As revealed that As toxicity accelerated the antioxidant defense mechanism [15]. However, 20 mg L^−1^ As resulted in oxidative destruction in the plant. Similar trends for electrolyte leakage and lipid peroxidation have been reported in Cu-, Cd-, and Pb-contaminated* Brassica napus* [16, 20, 23].

#### 3.2.4. Relative Water Contents (RWC) in* B. nivea* L

**Figure 5:**
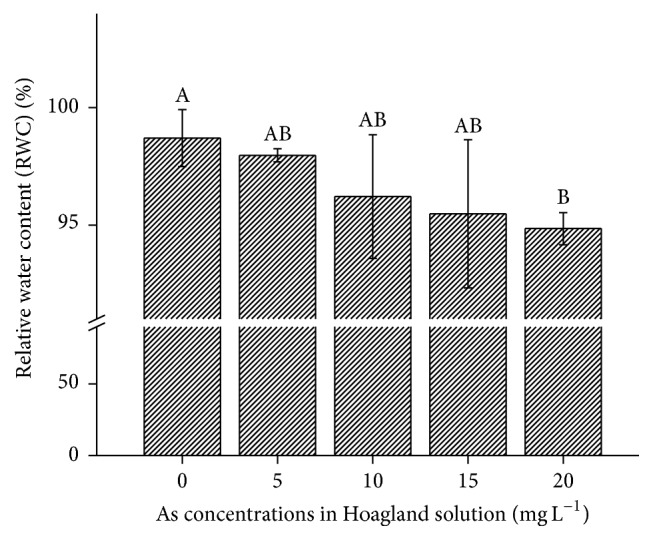
Effect of As application, on the RWC in* Boehmeria nivea* L. Values followed by different uppercase letters are significantly different at P < 0.05, for treatments. Different letters indicate significant differences between treatments for each parameter, at significant level of 0.05. Values followed by the same letters for each parameter are not significantly different at the 0.05 level (least significant difference). Values in the graph are mean (*n* = 3); error bars are standard deviation (SD).

As contamination reduced RWC in ramie, it was not significant (*P* > 0.05) (Figure 5). The reductions in RWC were 1.01–1.04-fold less than the control (1–4%) at 5–20 mg L^−1^ As. However, the greatest reduction in RWC (4%) was recorded at 20 mg L^−1^ As (Figure 5). The antioxidant defense mechanism enabled* B. nivea* to maintain tissue water potential and, therefore, cell turgor under the stress conditions [15]. Turgidity maintenance under stress leads to the maintenance of comparatively higher RWC under increasing As contamination (Figure 5). Similar trends for RWC and lipid peroxidation have been reported in Pb-contaminated ramie cultivars [15].

### 3.3. Arsenic (As) Concentration in* B. nivea* L

**Figure 6:**
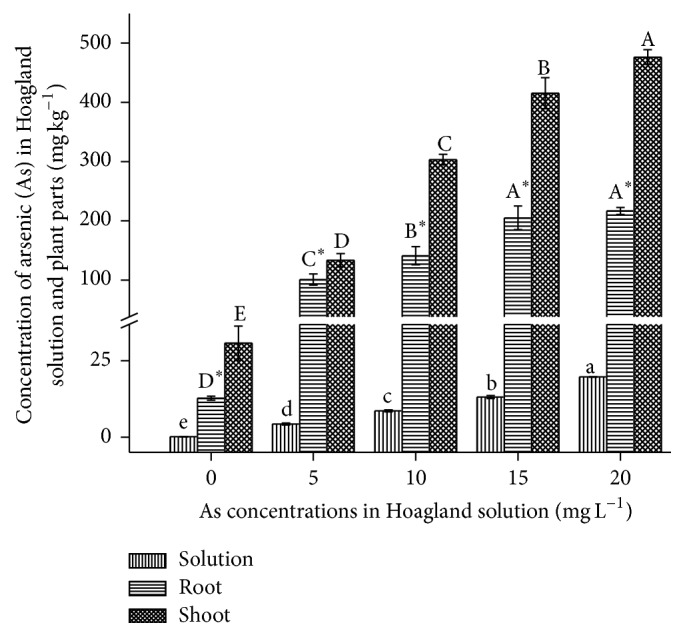
Effect of As application, on the As concentration in Hoagland solution and plant parts of* Boehmeria nivea* L. Values followed by different uppercase letters are significantly different at P < 0.05, for treatments. Values followed by different lowercase letters are significantly different at P < 0.05, for treatments. Values followed by different uppercase letters^*∗*^ are significantly different at P < 0.05, for treatments. Different letters indicate significant differences between treatments for each parameter, at significant level of 0.05. Values followed by the same letters for each parameter are not significantly different at the 0.05 level (least significant difference). Values in the graph are mean (*n* = 3); error bars are standard deviation (SD).

The As content in dried roots and shoots (leaves plus stem) of* B. nivea* increased significantly (*P* < 0.05) with increasing applied As (Figure 6), more so in the shoots than the roots. Arsenic (As) mostly accumulates in the aboveground parts of tolerant plants. The average amount of As remaining in the Hoagland solution at the end of the experiment was 89%.

The concentrations of As in the shoots and roots of* B. nivea* gradually significantly increased within a certain range, that is, 330–150 mg kg^−1^, respectively, compared to the control (94–75% > control). Shoot accumulation of As in* B. nivea* at 5 and 20 mg L^−1^ As was 4.0 and 15.5 times greater than the control, respectively, while in the roots the respective values were 8.0 and 17.0 times greater than the control, respectively. The average amount of As remaining in the Hoagland solution at 5, 10, 15, and 20 mg L^−1^ was 85, 86, 88, and 99% (i.e., 15, 14, 12, and 1% were absorbed by* B. nivea*), respectively. Thus, the performance of* B. nivea* improved with increasing addition of As. The mobilization of As from roots to leaves is the greatest threat to the food chain and the survival of life on Earth, but this would not occur in* B. nivea* because it annually sheds older leaves (which can be collected, removed, and/or recycled) and is a commercial fiber crop; hence, contamination of the food chain is avoided.

In contrast to our results, Otones et al. [33] recommended* Agrostis castellana* (Boiss. & Reut.),* Centaurea jacea* L.,* Eryngium campestre* L., and* Scirpus holoschoenus* L. for the stabilization of As in abandoned mining areas. According to this study, these plants showed low translocation factors, that is, underground [As] > aboveground [As]. In accordance with our results* Helichrysum oligocephalum*,* Hyoscyamus kurdicus*,* Nonea persica*,* Salvia syriaca* [34],* Rumex acetosella* L. [33],* Arundo donax* [35],* Isatis cappadocica*, and* Hesperis persica* [36] reportedly accumulate relatively high As concentrations in their shoots.

## 4. Conclusions

This study reports on the growth of* B. nivea* in As-contaminated hydroponic cultures up to 20 mg L^−1^. Arsenic contamination at high concentration, that is, 20 mg L^−1^, can inhibit growth, chlorophyll content, and SOD, CAT, and POD contents in the plant by inducing electrolyte leakage, lipid peroxidation, and reducing RWC. However, up to 15 mg L^−1^ As resulted in limited cellular oxidative damage in* B. nivea*. The plant accumulated higher As concentrations in shoots than roots and thus gave higher translocation factors. The ability to accumulate more metals in the stalk and leaves than roots is a positive indicator. The metabolic and biochemical processes in* B nivea* remain unaffected till 15 mg L^−1^ As, but at 20 mg L^−1^ stress-induced oxidative damage was apparent. This experiment suggests that* B. nivea* L. may extract a considerable amount of As; however a field-based study is needed to confirm these results.
